# Effect of Staple Age on DNA Origami Nanostructure Assembly and Stability

**DOI:** 10.3390/molecules24142577

**Published:** 2019-07-16

**Authors:** Charlotte Kielar, Yang Xin, Xiaodan Xu, Siqi Zhu, Nelli Gorin, Guido Grundmeier, Christin Möser, David M. Smith, Adrian Keller

**Affiliations:** 1 Technical and Macromolecular Chemistry, Paderborn University, Warburger Str. 100, 33098 Paderborn, Germany; 2 DNA Nanodevices Unit, Department Diagnostics, Fraunhofer Institute for Cell Therapy and Immunology IZI, 04103 Leipzig, Germany; 3 Institute of Biochemistry and Biology, Faculty of Science, University of Potsdam, 14476 Potsdam, Germany; 4 Peter Debye Institute for Soft Matter Physics, Faculty of Physics and Earth Sciences, University of Leipzig, 04103 Leipzig, Germany

**Keywords:** DNA origami, atomic force microscopy, stability, storage

## Abstract

DNA origami nanostructures are widely employed in various areas of fundamental and applied research. Due to the tremendous success of the DNA origami technique in the academic field, considerable efforts currently aim at the translation of this technology from a laboratory setting to real-world applications, such as nanoelectronics, drug delivery, and biosensing. While many of these real-world applications rely on an intact DNA origami shape, they often also subject the DNA origami nanostructures to rather harsh and potentially damaging environmental and processing conditions. Furthermore, in the context of DNA origami mass production, the long-term storage of DNA origami nanostructures or their pre-assembled components also becomes an issue of high relevance, especially regarding the possible negative effects on DNA origami structural integrity. Thus, we investigated the effect of staple age on the self-assembly and stability of DNA origami nanostructures using atomic force microscopy. Different harsh processing conditions were simulated by applying different sample preparation protocols. Our results show that staple solutions may be stored at −20 °C for several years without impeding DNA origami self-assembly. Depending on DNA origami shape and superstructure, however, staple age may have negative effects on DNA origami stability under harsh treatment conditions. Mass spectrometry analysis of the aged staple mixtures revealed no signs of staple fragmentation. We, therefore, attribute the increased DNA origami sensitivity toward environmental conditions to an accumulation of damaged nucleobases, which undergo weaker base-pairing interactions and thus lead to reduced duplex stability.

## 1. Introduction

DNA origami has become a widely employed technique for the rapid high-yield synthesis of arbitrary, yet well-defined, nanoscale shapes [1]. Since the first demonstration by Rothemund in 2006 [2], DNA origami nanostructures have found their way into many different fields of fundamental and applied research [3]. For instance, DNA origami nanostructures are currently employed as drug delivery vehicles [4,5,6,7,8], sensors [9,10,11,12], templates for the fabrication of nanoelectronic [13,14,15,16] and plasmonic devices [17,18,19,20,21], substrates for single-molecule studies [22,23,24,25,26,27], and masks in molecular lithography [28,29,30,31,32]. While all these applications crucially rely on an intact DNA origami shape, many of them subject the employed DNA origami nanostructures to rather harsh treatments. Consequently, interest in the effects that environmental and processing conditions exert on DNA origami structural integrity has spiked in the last few years [33,34,35,36,37,38,39,40,41,42,43,44,45].

Another issue that is becoming more and more relevant in the context of such applications is the long-term stability of the DNA origami nanostructures under relevant storage conditions [34,46,47]. While it has been established that, even under low-magnesium conditions, DNA origami nanostructures remain structurally intact over a period of several months when stored at 4 °C [34,47], their storage at higher temperatures may result in quick deterioration [46]. This issue can be circumvented by the lyophilization of the DNA origami nanostructures prior to storage [46].

While the aforementioned studies have focused on the storage of readily assembled DNA origami nanostructures, this work investigates the effect of the long-term storage of the employed staple strands on DNA origami assembly and stability. Atomic force microscopy (AFM) under liquid and dry conditions was employed to characterize the structural integrity of Rothemund triangles [2] assembled from different staple sets that have been stored at −20 °C for up to 43 months. In addition to comparing liquid and dry imaging conditions, we further employed different sample washing protocols to simulate different harsh processing conditions. We found that, while DNA origami assembly is largely unaffected by staple age, the assembled DNA origami triangles become gradually more sensitive toward harsh washing conditions as their staple age increased. Matrix-assisted laser desorption/ionization time-of-flight (MALDI-TOF) mass spectrometry investigations indicate that this is not a result of staple fragmentation but, rather, of damaged nucleobases. Finally, we present evidence that these staple age-related effects depend on the DNA origami superstructure.

## 2. Results and Discussion

In order to ensure comparability between staple sets of different age, all staple strands were mixed immediately after purchase, divided into 150 µL aliquots, and stored at −20 °C for 2 to 56 months. Therefore, all DNA origami samples were assembled from staple sets that have been frozen and thawed only once. For each DNA origami assembly, we used freshly defrosted tubes containing 10 µl of M13mp18 scaffold, aliquoted from a common stock solution, stored at −20 °C. To minimize the influence of scaffold preparation, the differently-aged staple sets were assembled using the same scaffold stock solution originating from the same preparation. Furthermore, the age of the scaffold stock solution at the time of use was always identical for each of the differently-aged staple sets used in one sample treatment protocol and comparable to the youngest staple set in each time series (below 8 months).

**Figure 1:**
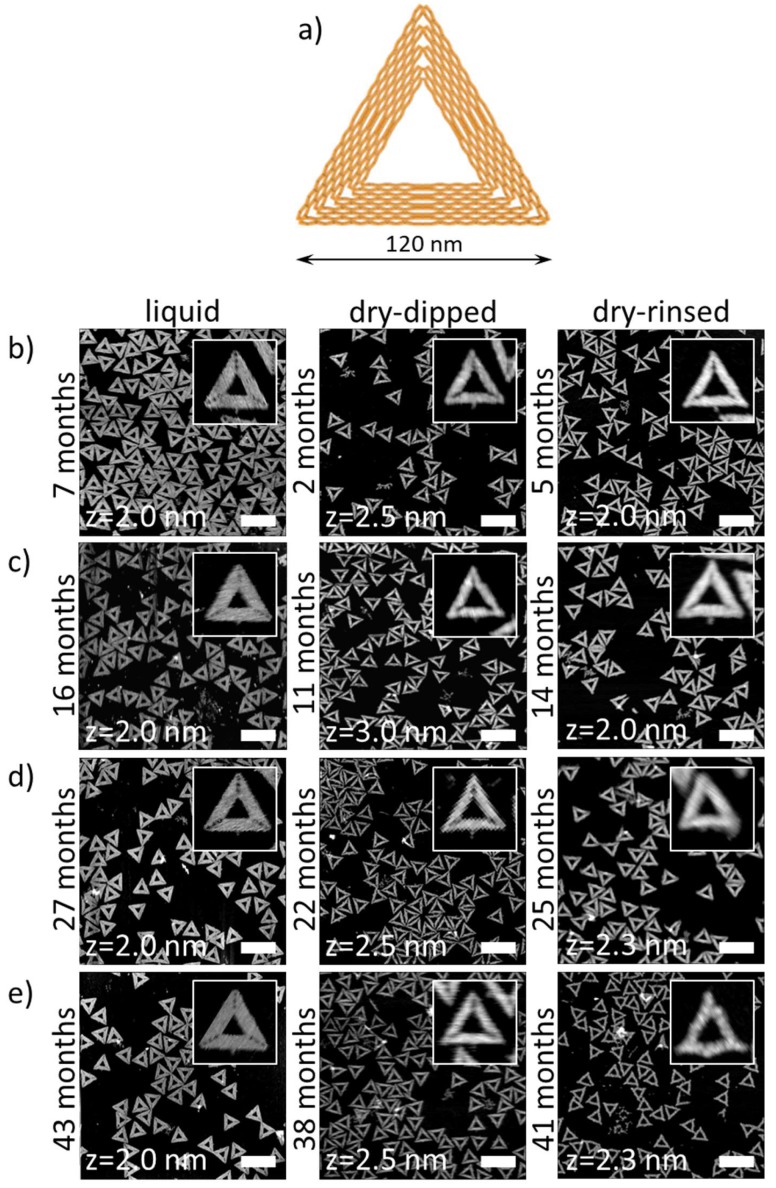
(**a**) Schematic illustration of the Rothemund triangle DNA origami. AFM images of DNA origami triangles assembled from staple sets aged for (**b**) 2–7 months, (**c**) 11–16 months, (**d**) 22–27 months, and (**e**) 38–43 months. Measurements were performed either in liquid (left column) or dry conditions after gently dipping the sample into water (central column) or after harsh rinsing (right column). Scale bars represent 250 nm. Height scales are given in the individual images. The insets show zooms of individual DNA origami triangles.

Figure 1b–e shows AFM images of Rothemund triangles assembled from staple sets of different ages. For comparison, a schematic representation of the duplex arrangement in the Rothemund triangle is given in Figure 1a. Ideally, AFM images of the assembled DNA origami should perfectly match the scheme. The AFM images in the left column of Figure 1b–e were recorded under liquid conditions in assembly buffer in order to assess any effects of staple age on the DNA origami assembly. Liquid imaging represents the least invasive AFM-based approach of analyzing DNA origami structural integrity at a nanometer resolution. Furthermore, it is frequently employed in DNA origami-based single-molecule studies [23,25,27,48,49,50,51]. As can be seen in the inset of Figure 1b (left), the Rothemund triangle assembled from seven-month-old staples shows all features expected from the scheme in Figure 1a. The three cavities in the corners of the triangle where the bridging staples connect the individual trapezoids are clearly visible, and even the seams in the centers of the trapezoids can be resolved. The overview image in Figure 1b (left) further suggests that the vast majority of Rothemund triangles are perfectly assembled, even though some small DNA origami fragments or broken/denatured triangles are occasionally observed. This general picture does not change significantly with increasing staple age. Even 43-month-old staples yield predominantly intact DNA origami triangles that show all the characteristic features of the design, indicating that staple age does not affect DNA origami assembly.

For many applications, the DNA origami nanostructures have to be dried after immobilization on a substrate surface. This is in order to quench certain reactions of attached chemical species [24] or, more frequently, to enable subsequent processing steps [13,15,22,26,28,29,31]. In all of these cases, the substrate needs to be washed with water in order to remove residual salt from the surface. The gentlest way to do this is by dipping the sample into pure water for a couple of seconds [24]. While this dipping may result in the dislocation and rearrangement of the adsorbed DNA origami nanostructures [52,53], it does not induce major damage of the Rothemund triangles [24].

The AFM image and the corresponding zoom of Rothemund triangles assembled from two-month-old staples shown in Figure 1b (center) reveal mostly intact DNA origami. Compared to liquid imaging, the triangular shapes appear less defined. This can be attributed both to drying-induced conformational alterations of the DNA duplexes in the DNA origami, such as B-A transitions, and minor shape distortions resulting from the removal of Mg^2+^ ions from the mica-DNA interface, which leads to a reduced strength of the interaction [54]. The latter is manifested, for example, in the decreased width of the trapezoids composing the triangles, which appear somewhat contracted or rolled up. Nevertheless, the cavities in the corners of the triangles can still be resolved, albeit not as separated cavities but as a groove. As for the measurements in solution, staple age does not seem to have a significant effect on DNA origami integrity. Even for 38-month-old staples, mostly intact triangles are observed after dipping.

For some applications of DNA origami nanostructures, gentle dipping of the substrate into water is not sufficient. This particularly concerns applications employing viscous buffer components or other molecular species that strongly adsorb to the substrate surface and need to be removed afterwards [28,33,36]. In such cases, the substrate surfaces are often rinsed with large amounts of water. The AFM image in Figure 1b (right) shows DNA origami triangles assembled from 5-month-old staples after such a rinsing treatment. The Rothemund triangles appear very similar to the ones subjected to dip-washing in Figure 1b (center), and there is no significant increase in the number of fragments and denatured DNA origami. Also, for Rothemund triangles assembled from 14-month-old staples (Figure 1c, right), this rather harsh treatment does not seem to impair DNA origami integrity.

At a staple age of 25 months, however, several Rothemund triangles with distorted shapes are observed in Figure 1d (right). The DNA origami in the corresponding inset appears somewhat swollen and has frayed edges with strongly rounded corners. It is also more difficult to identify the cavities in the corners of the triangles. These rinsing-induced shape distortions become even worse when 41-month-old staples are used for the DNA origami assembly. As can be seen in Figure 1e (right), the DNA origami still exhibits a roughly triangular shape. However, in this case, the trapezoids of many DNA origami are strongly deformed and bulging, and the cavities in the corners can no longer be identified.

**Figure 2:**
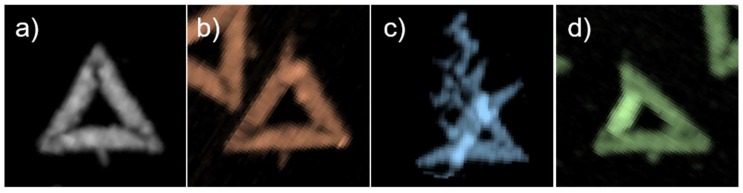
Representative AFM zooms of Rothemund triangles categorized as (**a**) intact, (**b**) broken, (**c**) denatured, and (**d**) deformed.

**Figure 3:**
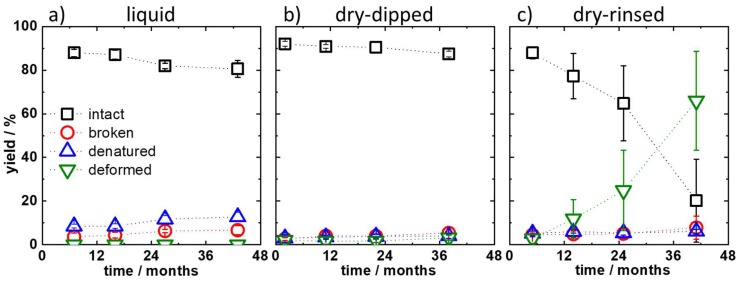
Yields of intact, broken, denatured, and deformed DNA origami depending on the age of the staple solution for (**a**) measurements in liquid, (**b**) measurements using the dry-dipped protocol, and (**c**) measurements using the dry-rinsed protocol.

In order to quantitatively evaluate the effects of staple age and treatment conditions on the structural integrity of the Rothemund triangles, the AFM images were analyzed by manually counting the fractions of intact, broken, deformed, and denatured DNA origami nanostructures. Figure 2 shows representative examples of these four categories. The results of the statistical analyses are shown in Figure 3 and Tables S1–S3 (see Supporting Information).

AFM imaging in liquid is the gentlest of the considered methods and, thus, expected to capture the solution state of the assembled DNA origami nanostructures. Nevertheless, Figure 3a shows that the yield of intact DNA origami decreased from ~88% at a staple age of 7 months to ~81% at a staple age of 43 months. This is due to the slight increase in the fractions of broken and denatured DNA origami with increasing the staple age. Interestingly, this increase occurred rather suddenly at a staple age between 16 and 27 months. These data indicate that staples older than this threshold age result in slightly more damaged DNA origami nanostructures. However, it is not clear from our measurements if staple age is correlated with lower assembly yields of intact DNA origami or whether the assembled DNA origami are more easily damaged during sample handling and purification.

The yields after dip-washing and drying the adsorbed DNA origami are given in Figure 3b. Only a very small decrease in the fraction of intact DNA origami, from 92 to 87%, is observed. Interestingly, dip-washing seems to result in a higher yield of intact DNA origami adsorbed onto the mica surface. This counterintuitive observation can be explained by the fact that broken and denatured DNA origami have a smaller surface area and, thus, a smaller contact area with the mica surface. Damaged DNA origami nanostructures are, therefore, more easily removed from the mica surface during dip-washing than intact ones, which superficially increases the measured yield of intact DNA origami.

In Figure 1, it can be observed that substrate rinsing leads to significant shape distortions and deformations in the adsorbed Rothemund triangles at a staple age of 25 months and older. The results of the statistical analyses shown in Figure 3c, however, show a significant decrease in the fraction of intact DNA origami already at a staple age of 14 months. The yield of intact DNA origami decreases from 88% at 5 months to only 76% at 14 months. At a staple age of 41 months, only 20% of the Rothemund triangles are still intact after rinsing. This is due to a strong increase in the yield of deformed DNA origami from only 3% at a staple age of 5 months to 66% at 41 months. Interestingly, the fractions of broken and denatured DNA origami are hardly affected by the staple age and remain in the range of 4 to 8%.

**Figure 4:**
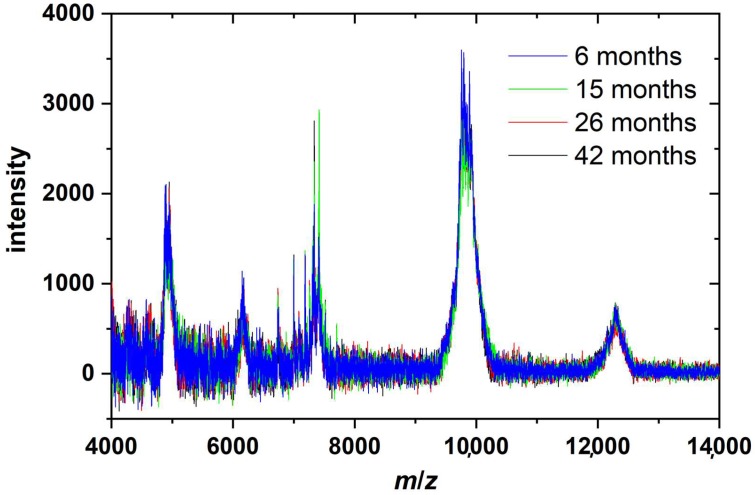
MALDI-TOF mass spectra of all four staple sets that differ in age from 6 to 42 months.

Our results indicate that the frozen staple strands are slowly degraded during storage. However, the fact that AFM imaging in liquid shows predominantly intact DNA origami even for the oldest staple sets implies that the DNA origami assembly itself is only mildly affected by staple age. Rather, the assembled DNA origami nanostructures appear to become more sensitive toward environmental conditions. Therefore, we speculate that the staple strands are not fragmented during storage, as this would result in the assembly of fewer intact DNA origami, but rather experience some sort of base damage that interferes with base pairing or stacking. In order to test this hypothesis, we analyzed the compositions of the complete staple mixtures using MALDI-TOF mass spectrometry. Figure 4 shows the spectra of all four staple sets that differ in age from 6 to 42 months. The DNA staple mixtures consisted of mainly three populations of strands based on their size. One peak (around 7400 Da) displays strands consisting of 22–25 nucleotides, a second peak (around 9800 Da) is from strands with 32 nucleotides, and a third peak (around 12,300 Da) shows strands consisting of 40 nucleotides. Peaks corresponding to the half-mass of the three main peaks (approximately 6100, 4900, and 3700 Da (not shown)) represent the doubly-ionized species. All spectra look identical regardless of staple age, and there is no apparent shift of individual peaks towards lower masses. Thus, any degradation or other fracturing of whole staple strands can be excluded.

**Figure 5:**
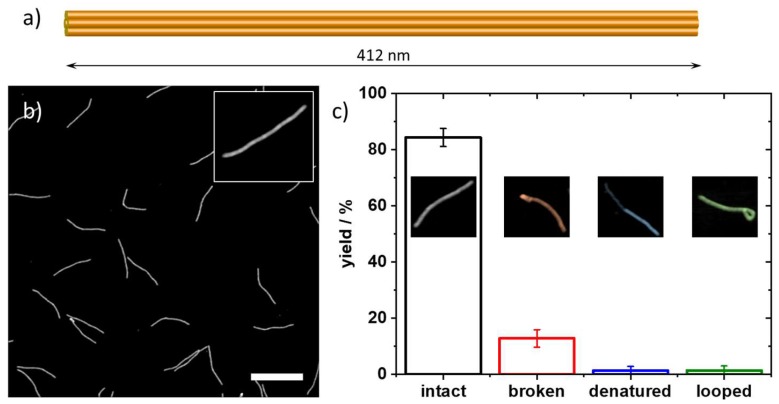
(**a**) Schematic illustration and (**b**) AFM image of DNA origami 6HBs assembled from 56-month-old staples after rinsing. The scale bar and height scale are 500 and 2.0 nm, respectively. The inset shows a zoom of an individual 6HB. (**c**) Corresponding yields of intact, broken, denatured, and looped DNA origami 6HBs. The insets show examples of the individual categories. Scaffold age at the time of use was one month.

Finally, we investigated whether these staple age-related effects also depend on the DNA origami superstructure. The Rothemund triangle is designed on a square lattice and is thus highly strained. Furthermore, its open, sheet-like structure offers no additional architectural stability apart from direct connections via crossovers to the neighboring helices. These two design factors may render it more susceptible to washing-induced damage than other DNA origami nanostructures. Therefore, we also subjected DNA origami six-helix bundles (6HBs) assembled from 56-month-old staples to the rinsing treatment and evaluated the effect of this using AFM. As can be seen in Figure 5, the 6HBs turn out to be much more robust than the Rothemund triangles and barely show any damage, with about 85% of the 6HBs remaining intact. This agrees with previous observations that these particular DNA origami nanostructures show extraordinarily high stability under denaturing conditions [34,38].

## 3. Materials and Methods

### 3.1. Preparation and Storage of the Staple Strands

Immediately upon delivery, the freshly synthesized staple strands (Metabion, Planegg, Germany), dissolved in pure water at 100 µM concentrations, were mixed at equal concentrations to yield the complete staple mixture. This stock solution was then divided into 150 µL aliquots and stored at −20 °C in the dark.

### 3.2. DNA Origami Assembly and Purification

The Rothemund triangles and DNA origami 6HBs were assembled from 208 and 170 staple strands, respectively, using the 7249-nt long M13mp18 genome as a scaffold. Assembly was performed in 1× TAE (Tris Acetate-EDTA, Carl Roth, Karlsruhe, Germany) containing 10 mM MgCl_2_ (Sigma-Aldrich, St. Louis, MO, USA) at a 10-fold excess of staples to scaffolds. Hybridization was carried out in a Thermocycler Primus 25 advanced (PEQLAB, Erlangen, Germany) by heating the sample to 80 °C and subsequently cooling it to room temperature over a time course of 90 min. The samples were then purified of excess staples with a 1× TAE/MgCl_2_ buffer by spin filtering using Amicon Ultra-0.5 mL Centrifugal Filters with 100 kDa molecular weight cut-off (Merck Millipore, Burlington, MA, USA), as previously described [34]. The DNA origami concentration was determined by UV/Vis absorption using an IMPLEN Nanophotometer (München, Germany) and ranged from 12 to 16 nM, independent of staple age. The DNA origami concentration was subsequently adjusted to 3 nM for all experiments.

### 3.3. Preparation of DNA Origami Samples for AFM Analysis

*Liquid.* 10 μL of the 3 nM DNA origami solution were pipetted onto a freshly cleaved mica surface in a liquid cell and incubated for 1 min. Subsequently, the cell was filled with 1 mL of 1× TAE/MgCl_2_.

*Dry-dipped.* 10 μL of the 3 nM DNA origami solution were incubated for 1 min on freshly cleaved mica. The mica substrate was then vertically dipped into pure water for 10 s and blown dry with a stream of ultra-pure air at an angle of 45° with respect to the substrate surface.

*Dry-rinsed.* 10 μL of the 3 nM DNA origami solution were incubated for 1 min on freshly cleaved mica. Using a pipette, the mica substrate was then rinsed five times with 2 mL of pure water at an angle of 45° with respect to the substrate surface. Then, the sample was blown dry with a stream of ultra-pure air at an angle of 45° with respect to the substrate surface.

### 3.4. AFM Imaging

AFM imaging in air was carried out using a JPK Nanowizard ULTRA Speed (Berlin, Germany), an Agilent 5100, and an Agilent 5500 AFM (Santa Clara, CA, USA), operated in intermittent contact mode. AFM imaging in liquid was carried out using a JPK Nanowizard ULTRA Speed. For measurements under dry and liquid conditions, HQ-NSC18/AlBS (MikroMasch, Sofia, Bulgaria) and USC-F0.3-k0.3 cantilevers (NanoWorld, Neuchâtel, Switzerland) were used, respectively.

### 3.5. Determination of Yields

In this section, the general procedure for determining the yields given in Tables S1–S3 is described. For dry imaging, two independent samples were analyzed for each staple age. For liquid imaging, only one sample was analyzed for each staple age. Five to ten AFM images were recorded for each staple age. For each recorded AFM image, the different yields were determined by manual counting. Final yields were obtained by finding the average of the yields obtained for the individual AFM images, with standard deviations as a measure of error. This is exemplified in Table 1 for the case of 38-month-old staple strands and imaging under dry conditions after dip-washing. The determined yields and numbers of DNA origami analyzed for all conditions are listed in Tables S1–S3.

### 3.6. MALDI-TOF Mass Spectrometry

For the mass spectrometry analysis, an Autoflex Speed mass spectrometer (Bruker Daltonik, Bremen, Germany) was used in linear positive mode. At first, 0.5 µL of 3-HPA matrix (half-saturated, dissolved in water/acetonitrile (50:50) with 10 mg/mL diammonium hydrogen citrate) were spotted on the AnchorChip target and dried at room temperature. Then, 0.5 µL of the DNA staple mixture were spotted and dried at room temperature. Before measurement, the machine was calibrated using the Oligonucleotide Calibration Standard (Bruker Daltonik, Bremen, Germany).

## 4. Conclusions

In summary, we investigated the effect of staple age on DNA origami assembly and stability using AFM under liquid and dry conditions. DNA origami assembly is only mildly affected by staple age, yielding approximately 80% intact Rothemund triangles when using a staple mixture stored for 43 months at −20 °C. However, the assembled Rothemund triangles become more sensitive toward environmental conditions with increasing staple age. In particular, the older the employed staples, the more deformed triangles are observed in AFM images recorded after extensive sample washing and drying. At a staple age of 41 months, this rinsing treatment results in 66% of the DNA origami showing significant shape distortions.

We could not detect any evidence of staple fragmentation in the aged staple mixtures using MALDI-TOF mass spectrometry, and we attribute this increased DNA origami sensitivity to an accumulation of damaged nucleobases which undergo weaker base-pairing interactions, resulting in reduced duplex stability. The mechanical forces acting on the adsorbed Rothemund triangles during rinsing, in combination with the depletion of stabilizing Mg^2+^ salt bridges, then lead to the dehybridization of some particularly weak staple strands from the scaffold and the subsequent rearrangement of the remaining duplexes within a given DNA origami. This finally results in the observed shape distortions. Unfortunately, the exact nature of the storage-induced base damage is very hard to assess because of the experimental complications arising when analyzing a mixture of approximately 200 non-purified oligonucleotides with different sequences and lengths. However, the different reaction rates displayed by the different types of spontaneously occurring nucleobase damage may provide some indication of the predominant type of damage. For instance, the hydrolytic deamination of cytosine in single-stranded DNA occurs with a half-life of about 200 years, while deamination of the other bases is much slower [55]. Hydrolytic depurination in single-stranded DNA occurs at similar half-lives of around 100 years, while the loss of pyrimidine bases is slower by several orders of magnitude [55]. Because of these long reaction times, hydrolytic deamination and base loss appear rather unlikely candidates in the formation of excessive nucleobase damage in frozen oligonucleotides over a time course of a few years. On the other hand, OH radical-driven oxidative base damage, such as the generation of 8-oxo-guanine or ring-opened lesions in guanine and thymine, occurs much faster with half-lives of the order of hours [55]. Furthermore, oxidative base damage has been shown to result in the destabilization of the DNA duplex [56,57]. Therefore, we assume that oxidative base damage is the origin of the observed increase in DNA origami sensitivity.

Our results reveal a complex interplay between staple storage, DNA origami treatment conditions, and the DNA origami superstructure. If the assembled DNA origami nanostructures are employed only in liquid under mild environmental conditions and using appropriate buffers [34], staple age will not play a significant role at all. In the case of harsh environmental conditions, such as excessive sample rinsing (as demonstrated here), exposure to denaturing buffer conditions [33,36], or elevated temperatures, the structural integrity of the DNA origami may be seriously impaired if staples older than a few months are used. As we have demonstrated, however, the degree of staple age-induced damage may drastically depend on the DNA origami superstructure, and this needs to be evaluated individually for each DNA origami design under the relevant conditions. Finally, we would like to stress that the above experiments were conducted with staple mixtures dissolved in pure water that have been frozen and thawed only once. Repeated freezing and thawing cycles may induce more serious damage and have more devastating effects on DNA origami assembly and structural stability than observed here. On the other hand, the use of buffers instead of pure water may reduce the negative effects of long-term storage [58]. Furthermore, since oxidative base damage appears to be the most likely origin of the observed increase in DNA origami sensitivity, the addition of antioxidants may also improve the quality of the staple strands after long-term freeze storage.

## Figures and Tables

**Table 1:** Number of analyzed DNA origami triangles (N_(DNA origami)_) for each of the eight AFM images recorded after dip-washing a sample assembled from 38-month-old staples and the corresponding yields of intact, broken, deformed, and denatured DNA origami in %.

AFM Image	N_(DNA origami)_	Intact DNA Origami (%)	Broken DNA Origami (%)	Denatured DNA Origami (%)	Deformed DNA Origami (%)
1	524	89.5	4.2	4.8	1.5
2	518	88.2	6.2	4.1	1.5
3	444	86.0	5.0	5.2	3.8
4	515	86.8	4.8	6.0	3.1
5	491	88.0	5.7	3.1	3.3
6	526	85.2	7.0	3.4	4.4
7	552	88.2	4.5	3.1	4.2
8	554	87.5	6.0	2.7	3.8
**Sum**	**4124**	**87.4 ± 1.4**	**5.4 ± 1.0**	**4.0 ± 1.2**	**3.2 ± 1.1**

## Supplementary Materials

The following are available online, Tables S1–S3: yields for the Rothemund triangles obtained from the evaluation of the individual AFM images recorded under liquid and dry conditions, Table S4–S6: *p*-values of the individual yields for the Rothemund triangles, Table S7: yields for the 6HBs obtained from the evaluation of the individual AFM images, Figures S1–S13: additional AFM images.
