# Supplementary material for: Effect of Staple Age on DNA Origami Nanostructure Assembly and Stability
Source: Molecules. 2019 Jul 16;24(14):2577. doi: 10.3390/molecules24142577 (PMC6680526; doi:10.3390/molecules24142577)
Supplement: Supplementary file 1 [file molecules-24-02577-s001.pdf]

molecules

Supporting information

Effect of staple age on DNA origami nanostructure assembly and stability

Charlotte Kielar [1], Yang Xin [1], Xiaodan Xu [1], Siqi Zhu [1], Nelli Gorin [1], Guido Grundmeier [1], Christin Möser [2,3], David M. Smith [2,4], and Adrian Keller [1,*]

[1] Technical and Macromolecular Chemistry, Paderborn University, Warburger Str. 100, 33098 Paderborn, Germany
[2] DNA Nanodevices Unit, Department Diagnostics, Fraunhofer Institute for Cell Therapy and Immunology IZI, 04103 Leipzig, Germany
[3] Institute of Biochemistry and Biology, Faculty of Science, University of Potsdam, 14476 Potsdam, Germany
[4] Peter Debye Institute for Soft Matter Physics, Faculty of Physics and Earth Sciences, University of Leipzig, 04103 Leipzig, Germany
* Correspondence: adrian.keller@uni-paderborn.de

Table S1. Yields (in %) of intact, broken, and denatured Rothemund triangles assembled from staples of different age obtained by AFM in liquid.

liquid				
**months**	**intact**	**broken**	**denatured**	**N(DNA origami)**
7	88.0 ± 1.7	3.3 ± 1.7	8.5 ± 0.8	727
16	87.2 ± 2.1	4.3 ± 1.4	8.5 ± 1.0	508
27	82.0 ± 1.2	6.3 ± 0.8	11.7 ± 1.7	611
43	80.6 ± 3.9	6.7 ± 2.0	12.7 ± 2.1	844

Table S2. Yields (in %) of intact, broken, denatured, and deformed Rothemund triangles assembled from staples of different age obtained by AFM in the dry state after dip-washing.

dry-dipped					
**months**	**intact**	**broken**	**denatured**	**deformed**	**N(DNA origami)**
2	92.1 ± 1.2	2.7 ± 0.7	2.9 ± 1.7	2.1 ± 1.6	1967
11	90.9 ± 0.9	4.0 ± 1.2	3.4 ± 1.0	1.6 ± 1.4	3730
22	90.6 ± 2.3	4.0 ± 1.0	3.8 ± 0.9	1.7 ± 0.7	3728
38	87.5 ± 1.4	5.4 ± 1.0	4.0 ± 1.1	3.2 ± 1.1	4140

Table S3. Yields (in %) of intact, broken, denatured, and deformed Rothemund triangles assembled from staples of different age obtained by AFM in the dry state after rinsing.

dry-rinsed					
**months**	**intact**	**broken**	**denatured**	**deformed**	**N(DNA origami)**
5	87.8 ± 2.9	4.4 ± 0.8	5.1 ± 1.1	2.7 ± 2.1	2769
14	76.3 ± 9.5	5.0 ± 0.0	6.5 ± 1.2	12.2 ± 8.6	1880
25	64.8 ± 17.2	5.0 ± 2.3	5.4 ± 1.0	24.8 ± 18.4	2353
41	20.1 ± 19.1	7.8 ± 5.3	6.1 ± 1.0	66.0 ± 22.7	2353

Table S4. *p*-values of the yields of intact, broken, and denatured Rothemund triangles assembled from staples of different age obtained by AFM in in liquid. The *p*-values were determined using Student's t-test (two-tailed distribution, homoscedastic) with regard to the samples assembled from the youngest staple set (7 months).

liquid			
**months**	**intact**	**broken**	**denatured**
16	0.538908	0.457942	0.966009
27	0.000221	0.012058	0.004937
43	0.003832	0.024501	0.002318

Table S5. *p*-values of the yields of intact, broken, denatured, and deformed Rothemund triangles assembled from staples of different age obtained by AFM in the dry state after dip-washing. The *p*-values were determined using Student's t-test (two-tailed distribution, homoscedastic) with regard to the samples assembled from the youngest staple set (2 months).

dry-dipped				
**months**	**intact**	**broken**	**denatured**	**deformed**
11	0.043805	0.016803	0.449779	0.537363
22	0.113343	0.009389	0.232761	0.577788
38	0.000005	0.000015	0.154431	0.126168

Table S6. *p*-values of the yields of intact, broken, denatured, and deformed Rothemund triangles assembled from staples of different age obtained by AFM in the dry state after rinsing. The *p*-values were determined using Student's t-test (two-tailed distribution, homoscedastic) with regard to the samples assembled from the youngest staple set (5 months).

dry-rinsed				
**months**	**intact**	**broken**	**denatured**	**deformed**
14	0.005501	0.272474	0.031780	0.008865
25	0.002261	0.492995	0.667026	0.004497
41	0.0000001	0.094633	0.094889	0.000002

Table S7. Yields (in %) of intact, broken, denatured, and looped 6HB DNA origami assembled from 56 month-old staples obtained by AFM in the dry state after rinsing.

dry-rinsed				
**intact**	**broken**	**denatured**	**looped**	**$N_{(DNA\ origami)}$**
84.4 ± 3.2	12.8 ± 3.1	1.4 ± 1.5	1.4 ± 1.6	572

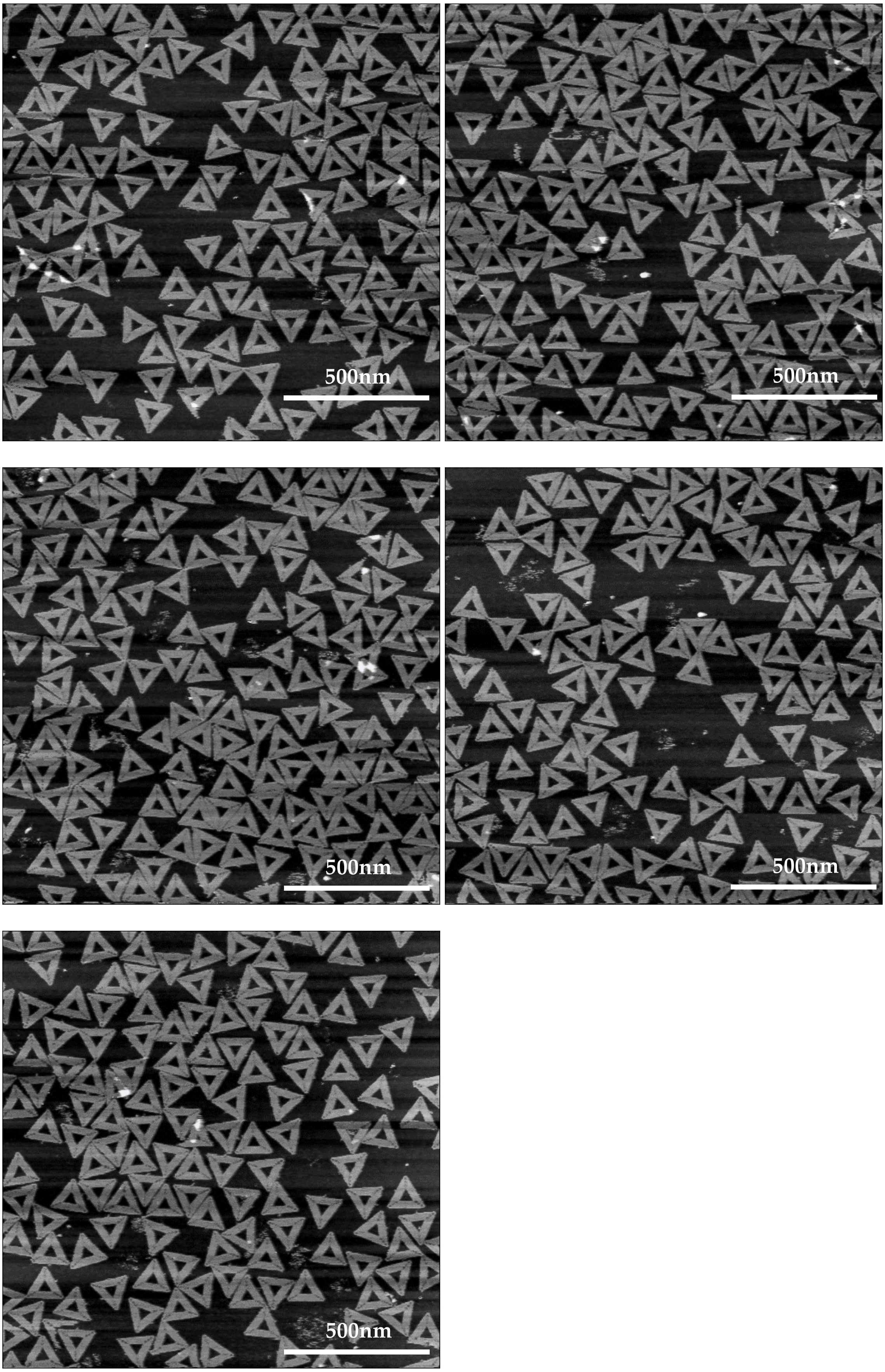

Figure S1. Additional AFM images of DNA origami triangles assembled from 7 month-old staples recorded in liquid.

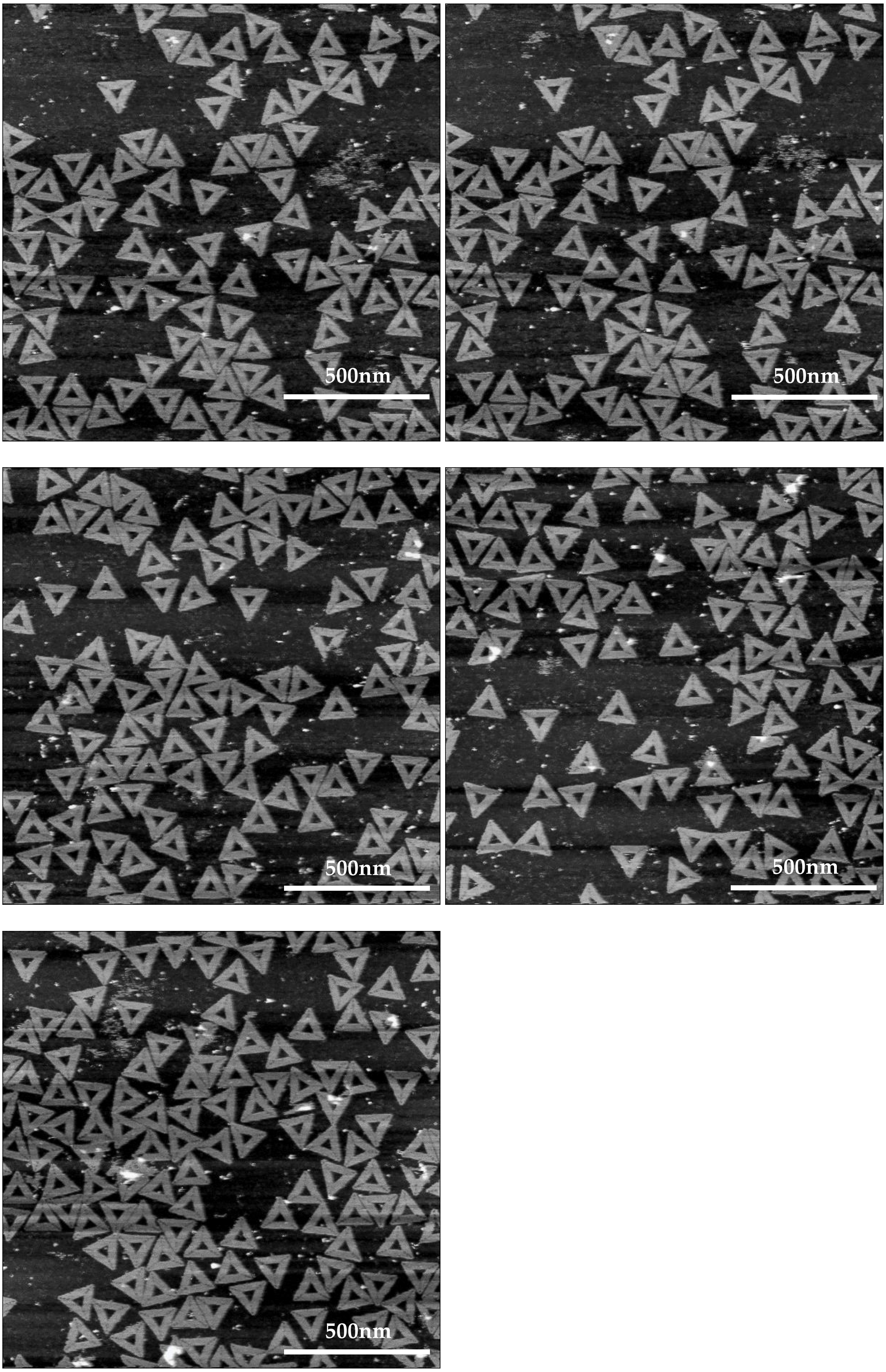

Figure S2. Additional AFM images of DNA origami triangles assembled from 16 month-old staples recorded in liquid.

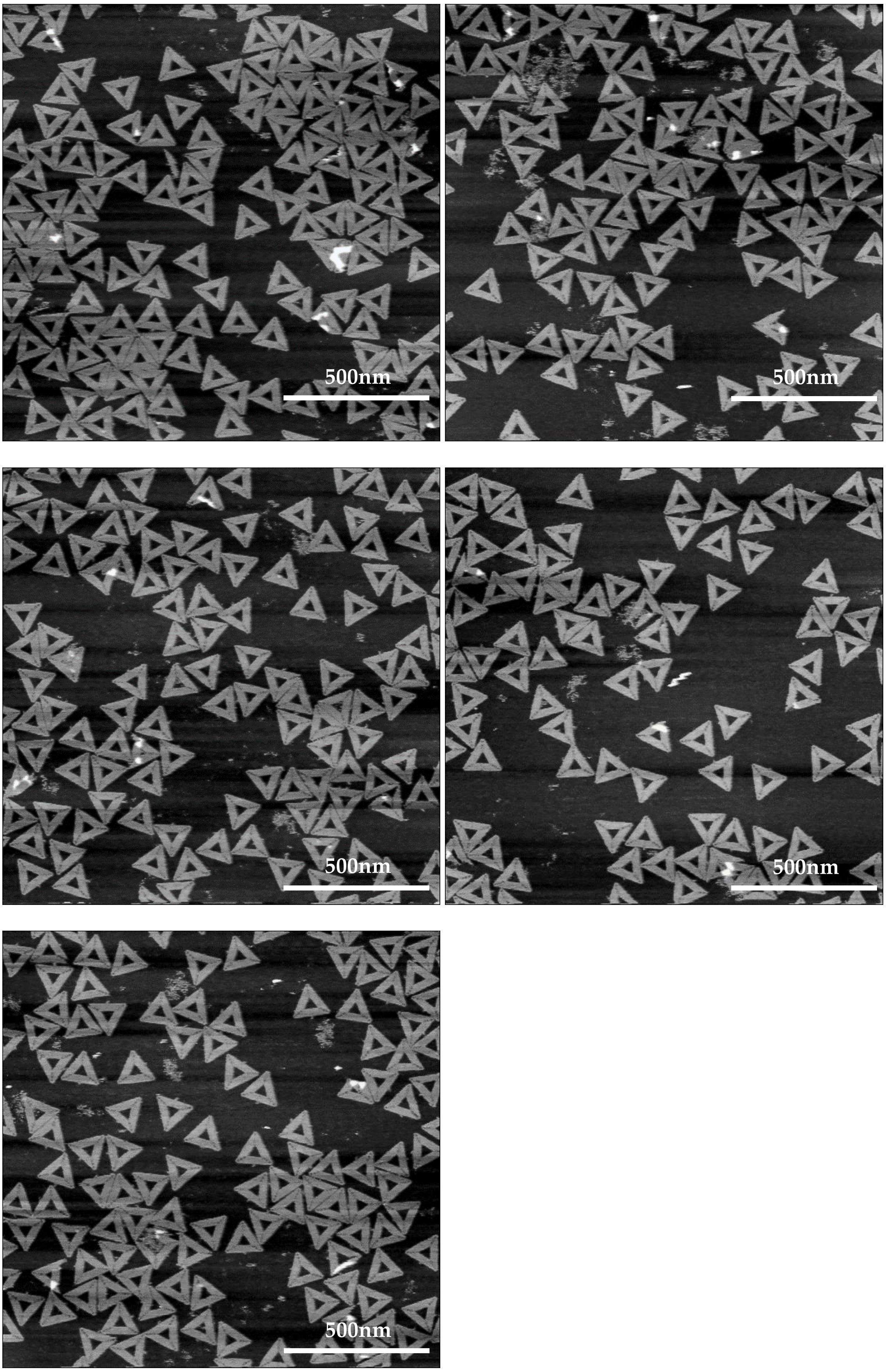

Figure S3. Additional AFM images of DNA origami triangles assembled from 27 month-old staples recorded in liquid.

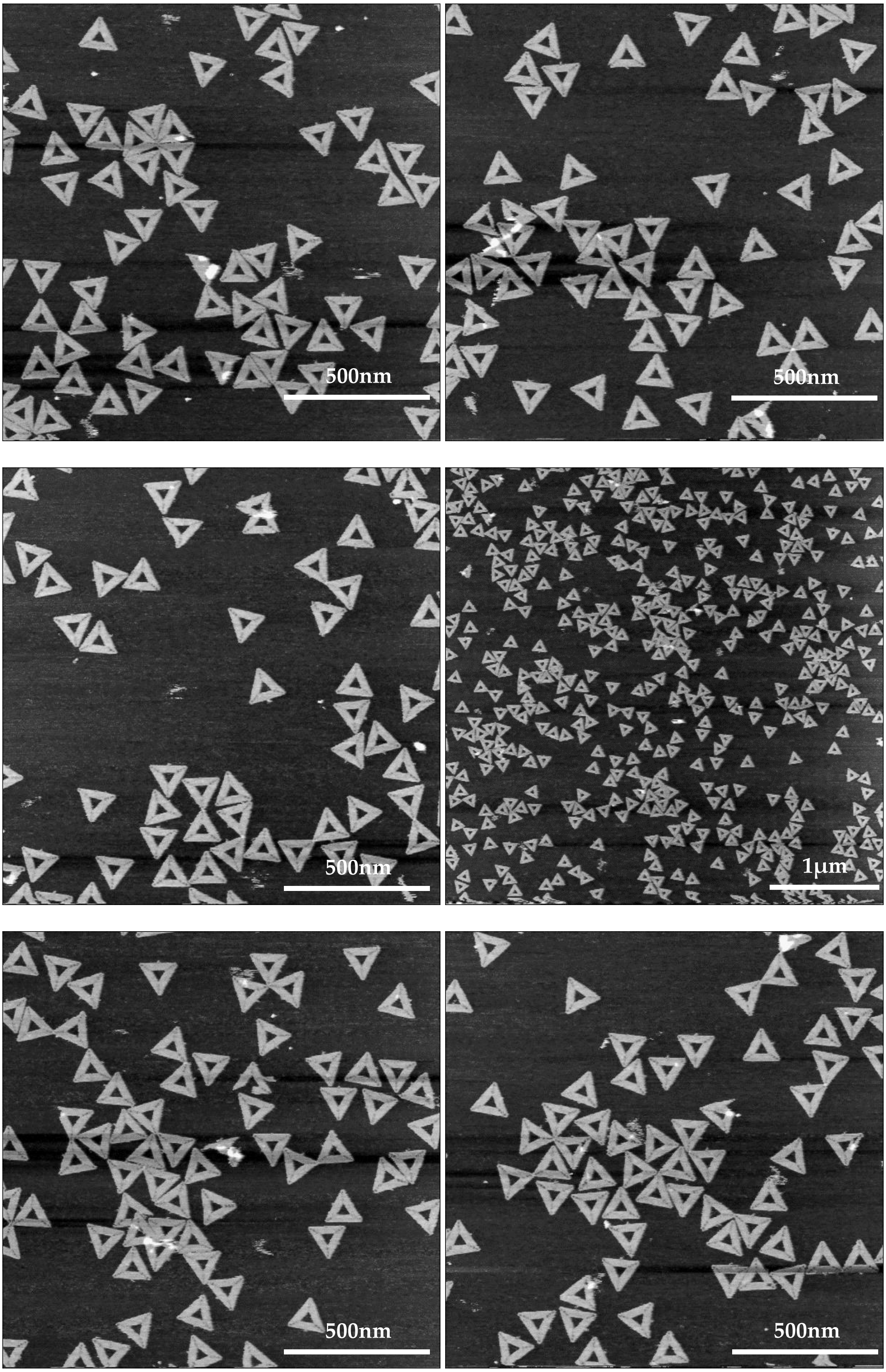

Figure S4. Additional AFM images of DNA origami triangles assembled from 43 month-old staples recorded in liquid.

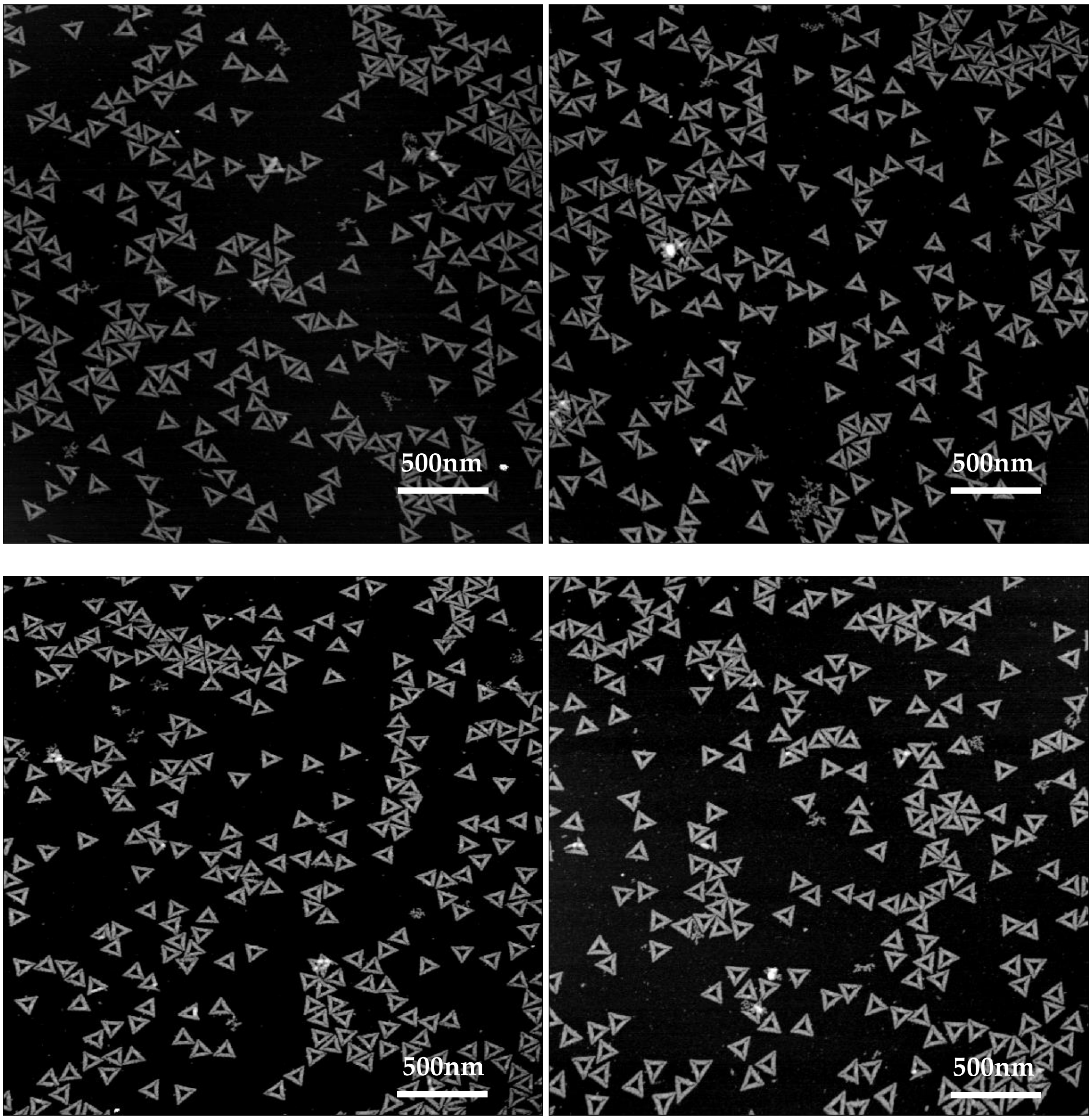

Figure S5. Additional AFM images of DNA origami triangles assembled from 2 month-old staples recorded after dip-washing.

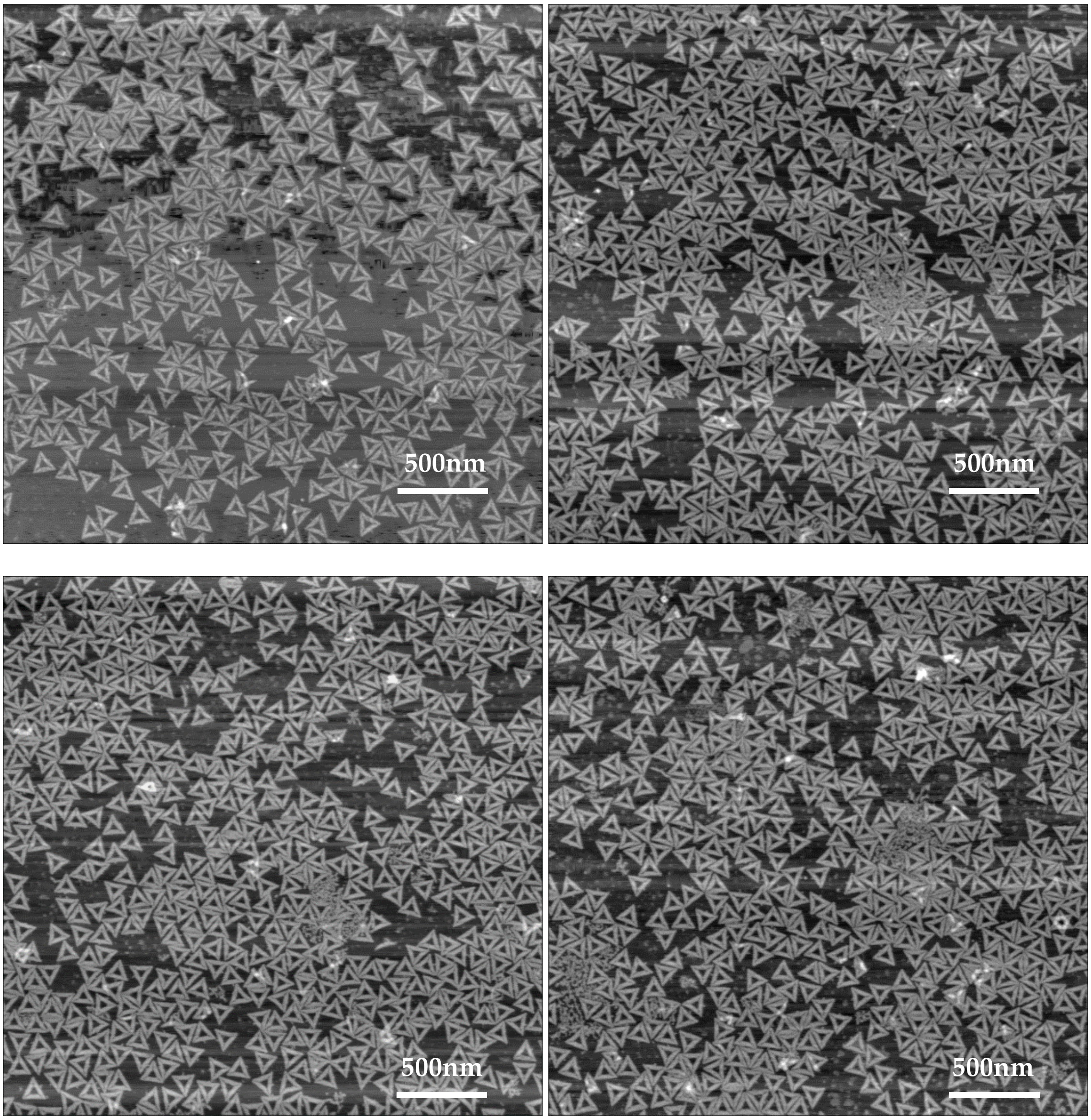

Figure S6. Additional AFM images of DNA origami triangles assembled from 11 month-old staples recorded after dip-washing.

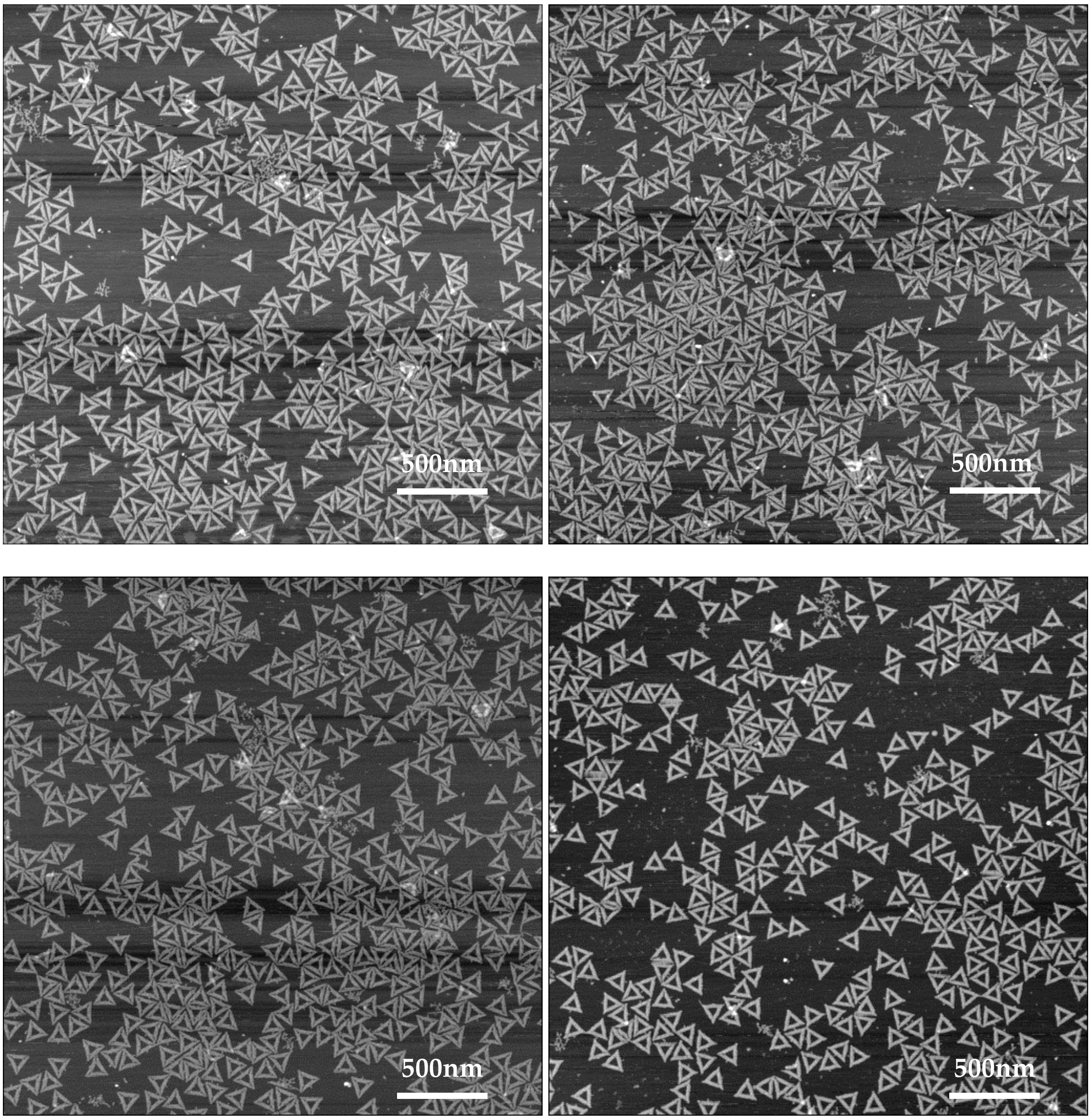

Figure S7. Additional AFM images of DNA origami triangles assembled from 22 month-old staples recorded after dip-washing.

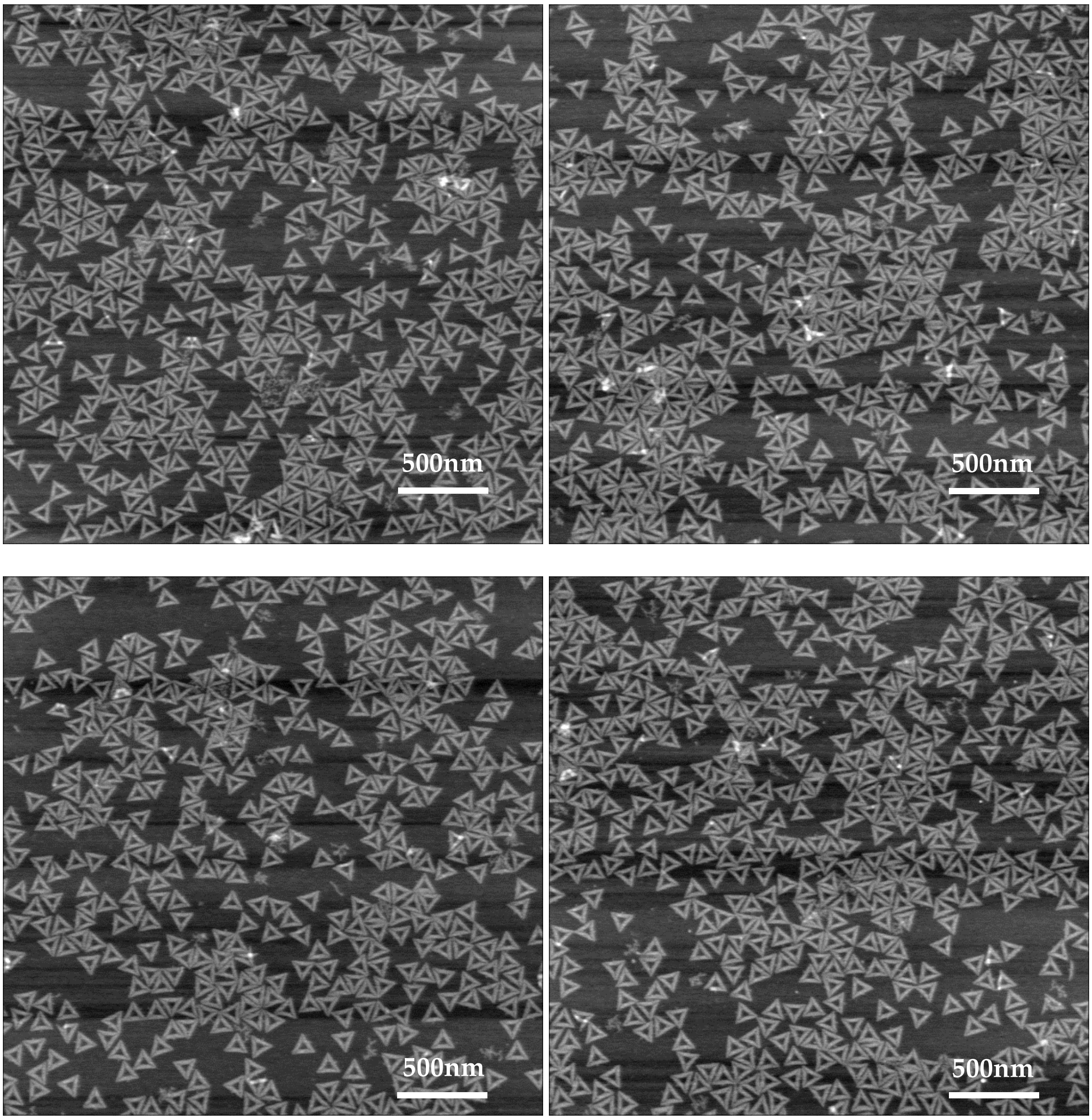

Figure S8. Additional AFM images of DNA origami triangles assembled from 38 month-old staples recorded after dip-washing.

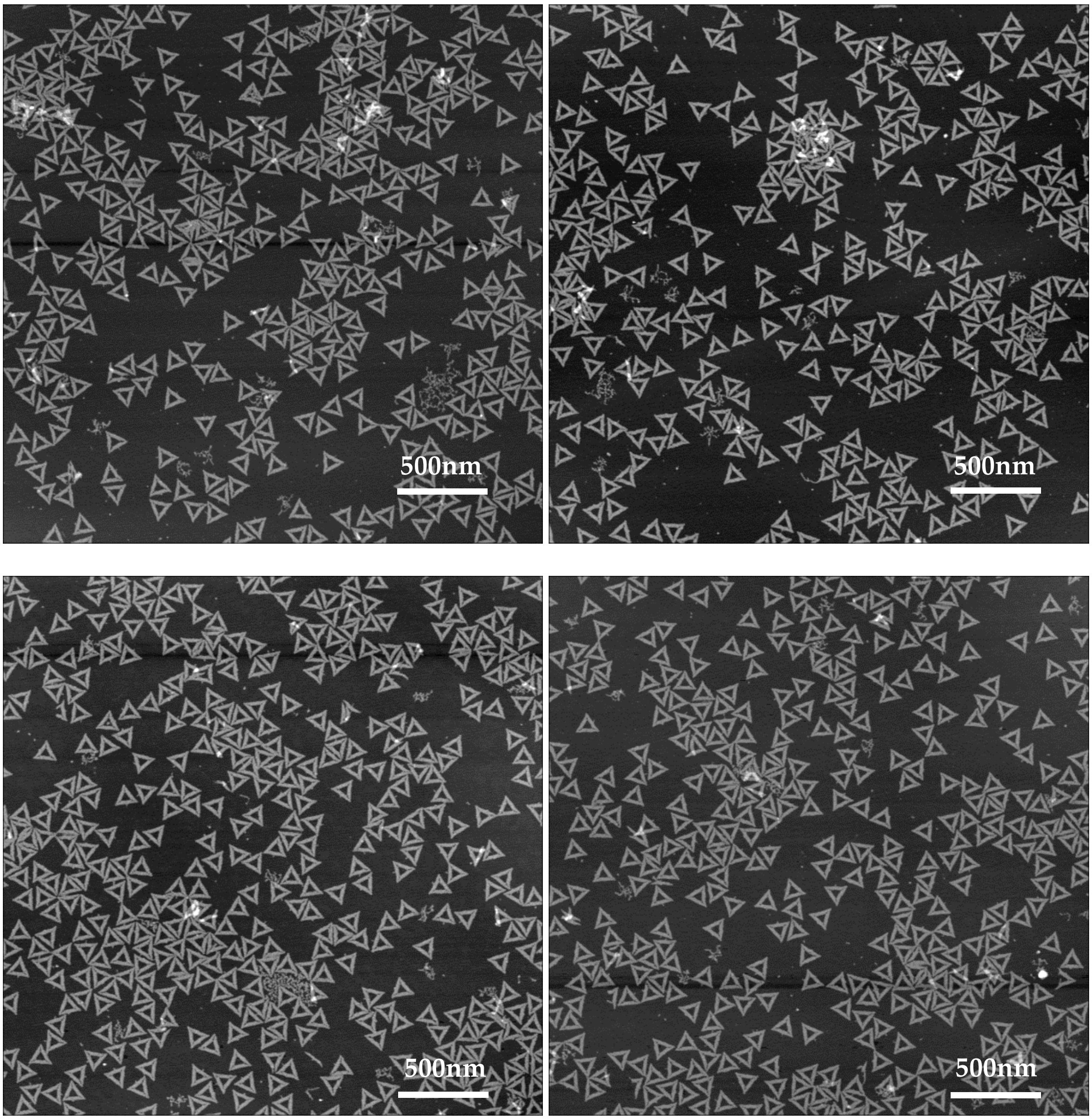

Figure S9. Additional AFM images of DNA origami triangles assembled from 5 month-old staples recorded after rinsing.

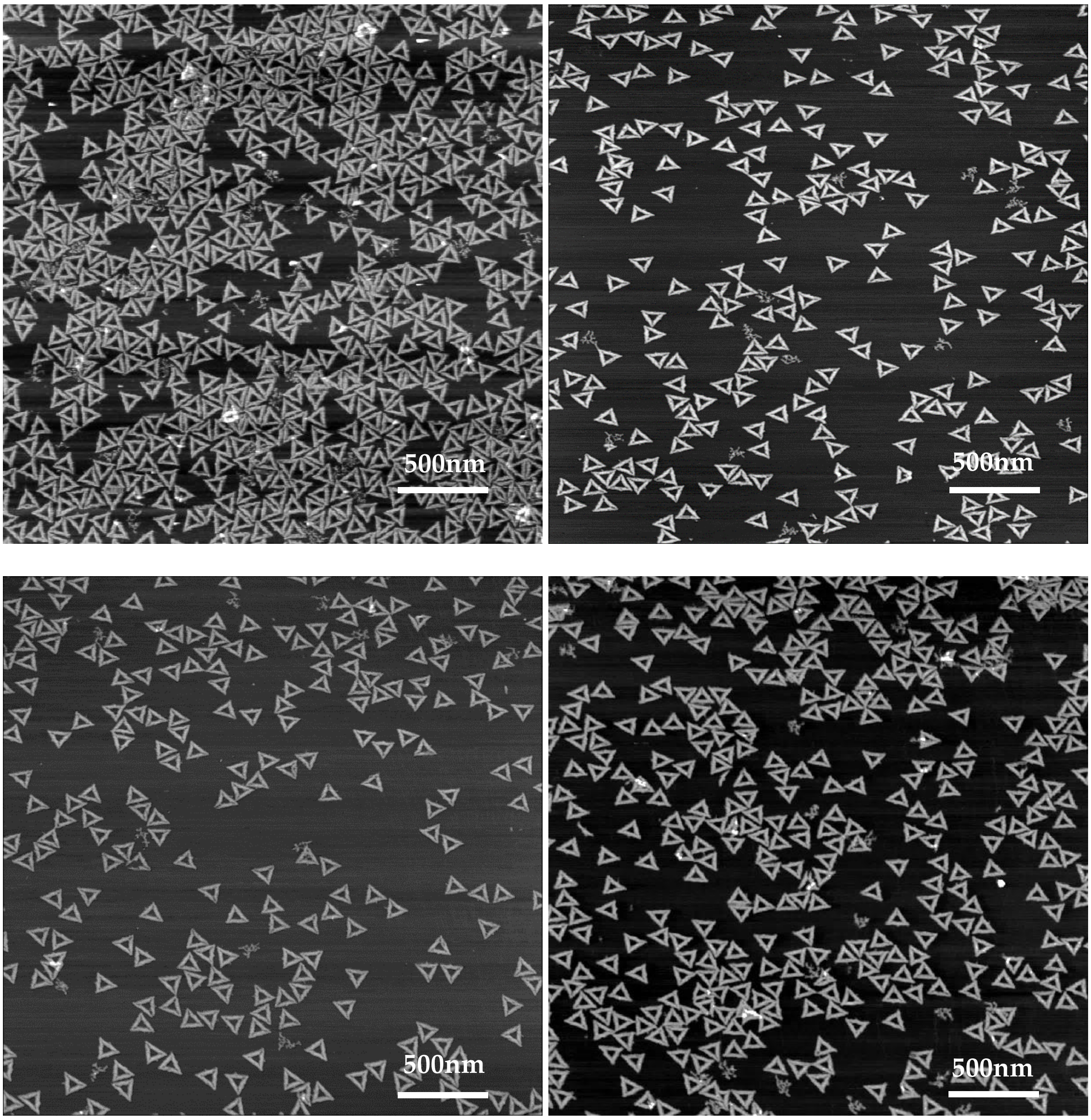

Figure S10. Additional AFM images of DNA origami triangles assembled from 14 month-old staples recorded after rinsing.

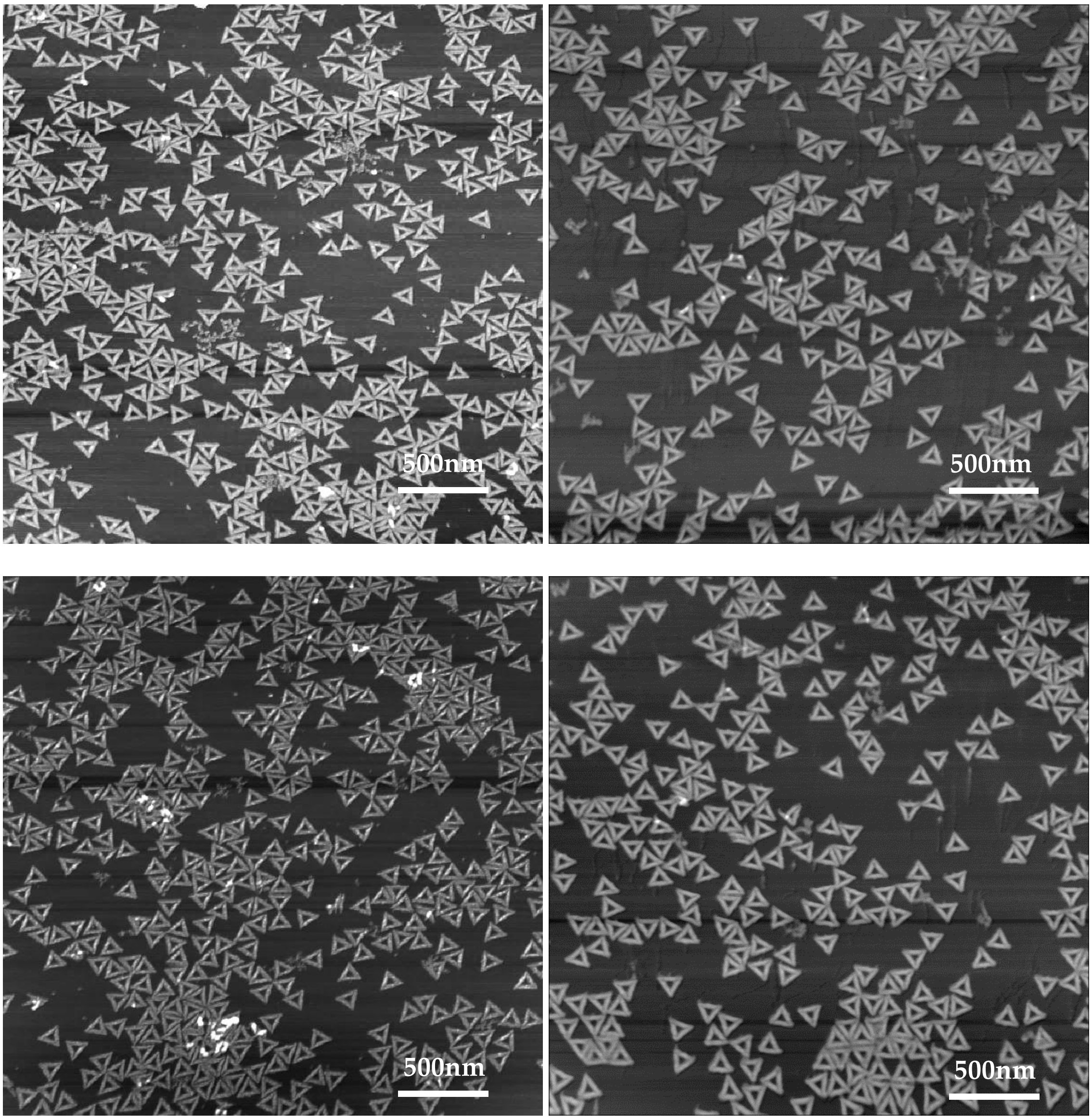

Figure S11. Additional AFM images of DNA origami triangles assembled from 25 month-old staples recorded after rinsing.

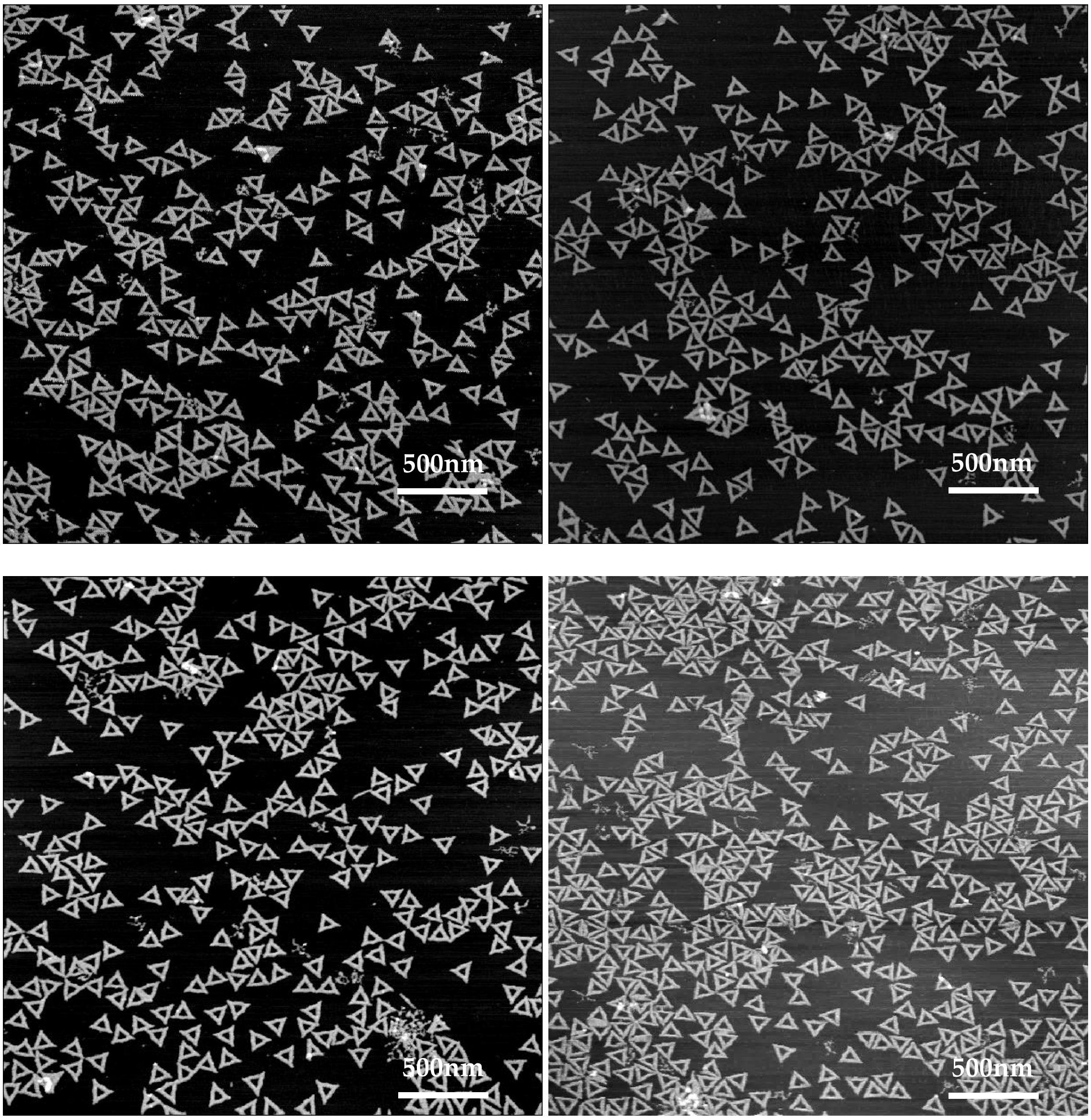

Figure S12. Additional AFM images of DNA origami triangles assembled from 41 month-old staples recorded after rinsing.

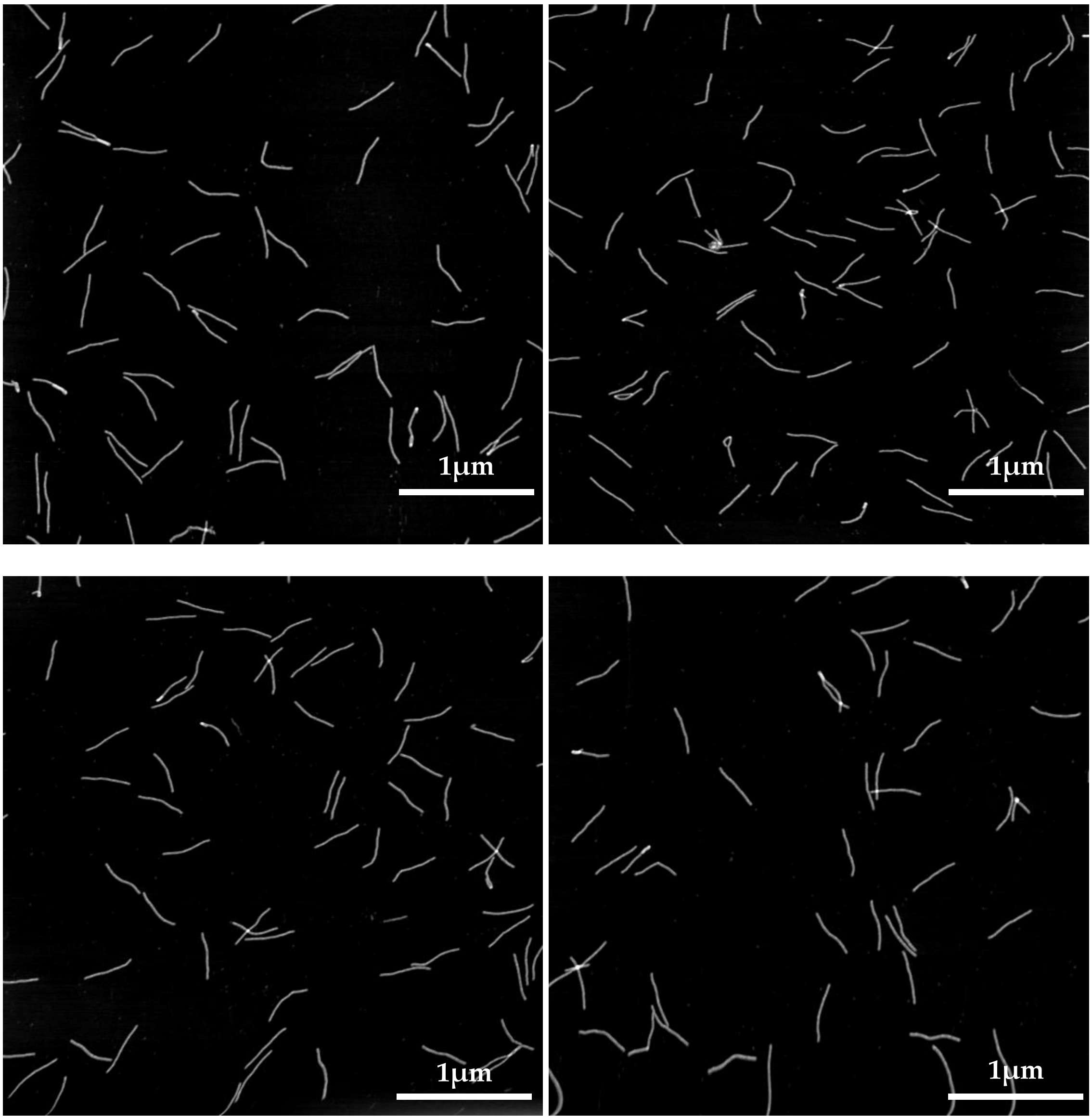

Figure S13. Additional AFM images of DNA origami 6HBs assembled from 56 month-old staples recorded after rinsing.
